# Fault Diagnosis Method for Wind Turbine Gearbox Based on Ensemble-Refined Composite Multiscale Fluctuation-Based Reverse Dispersion Entropy

**DOI:** 10.3390/e26080705

**Published:** 2024-08-20

**Authors:** Xiang Wang, Yang Du

**Affiliations:** 1 School of Energy and Power Engineering, Nanjing Institute of Technology, Nanjing 211167, China; 2 School of Electrical Engineering, Nanjing Institute of Technology, Nanjing 211167, China; y00450220746@njit.edu.cn

**Keywords:** fault diagnosis, wind turbine gearbox, noise reduction, fluctuation-based reverse dispersion entropy, least squares support vector machine

## Abstract

The diagnosis of faults in wind turbine gearboxes based on signal processing represents a significant area of research within the field of wind power generation. This paper presents an intelligent fault diagnosis method based on ensemble-refined composite multiscale fluctuation-based reverse dispersion entropy (ERCMFRDE) for a wind turbine gearbox vibration signal that is nonstationary and nonlinear and for noise problems. Firstly, improved complete ensemble empirical mode decomposition with adaptive noise (ICEEMDAN) and stationary wavelet transform (SWT) are adopted for signal decomposition, noise reduction, and restructuring of gearbox signals. Secondly, we extend the single coarse-graining processing method of refined composite multiscale fluctuation-based reverse dispersion entropy (RCMFRDE) to the multiorder moment coarse-grained processing method, extracting mixed fault feature sets for denoised signals. Finally, the diagnostic results are obtained based on the least squares support vector machine (LSSVM). The dataset collected during the gearbox fault simulation on the experimental platform is employed as the research object, and the experiments are conducted using the method proposed in this paper. The experimental results demonstrate that the proposed method is an effective and reliable approach for accurately diagnosing gearbox faults, exhibiting high diagnostic accuracy and a robust performance.

## 1. Introduction

Wind power generation is increasingly vital in the field of renewable energy owing to the swift advancement of new energy technologies [1]. Their operation and maintenance costs show an exponential growth trend as the installed capacity of wind turbines continues to increase. Gearboxes, which are among the most precise and costly components of wind turbines, represent a significant portion of the investment [2]. In order to minimize the economic losses caused by gearbox failures leading to unit shutdowns, timely warnings and an accurate identification of fault types in fault diagnosis technology become particularly important [3]. Fault diagnosis technology is one of the key factors for the stable development of wind power [4]. The collection of operational data from various key positions of wind turbine units is achieved through the utilization of state detection technology, which is then subjected to intelligent signal processing techniques to evaluate the overall condition of the units. The accurate identification of fault types during abnormal operation of the units provides reliable support for maintenance work. Fault diagnosis of wind power units generally includes three steps: signal processing [5], feature extraction [6], and fault identification [7].

Vibration signals collected from gearboxes in harsh environments are frequently contaminated by a significant amount of noise, which complicates the extraction of signal features. Therefore, it is crucial to perform time–frequency domain analyses on the signals [8]. The most commonly employed signal processing techniques include empirical mode decomposition (EMD) [9], variational mode decomposition (VMD) [10], and related improved methods. ICEEMDAN was proposed in 2014 [11] and is an improved algorithm of the complementary ensemble EMD (CEEMD) method, which reduces a lot of unnecessary components and reduces the pseudo-modalities to a large extent. ICEEMDAN is extensively utilized in signal processing, data analysis, image processing, and other related applications with the advantage of sufficient decomposition [12,13,14]. In 2023, Su et al. [15] were inspired by the natural frost growth mechanism and proposed the rime optimization algorithm (RIME). The RIME algorithm simulates the movement of delicate ice crystals for the purpose of developing algorithms through the modeling of interaction patterns between hard frost agents, exhibiting a high global search capability, fast convergence, and wide adaptability [16]. In this study, RIME is employed to optimize the number of realizations (NR) and the noise standard deviation (Nsd) of the added noise during ICEEMDAN decomposition.

The issue of filtering noise-containing and noise-free signals represents a significant challenge for the intrinsic modal function (IMF) components obtained by ICEEMDAN decomposition. Effective signals typically display a certain degree of periodicity or regularity, whereas noise often manifests as random or irregular phenomena [17]. Permutation entropy (PE) is highly sensitive to the intricacy of vibration signal temporal data sequences [18], enabling the capture of regular changes in the signals and the effective distinction between signal and noise components. In this study, PE is selected as the criterion for evaluating the noise-containing and noise-free signals in the IMF components. The threshold value has been set to 0.6 [19] and will determine whether the IMF component requires noise reduction.

The conventional approach to noise cancellation entails the utilization of filters for the reduction of signal noise by applying the spectral distribution law of signal and noise [20]. The Fourier transform is initially employed to transform the signal containing noise into the frequency domain [21]. Subsequently, a low-pass filter is utilized for the purpose of denoising. Wavelet transform (WT) exhibits excellent time–frequency localization characteristics, which have been extensively investigated in the context of signal denoising, resulting in highly promising outcomes [22]. It has emerged as a pivotal approach in signal denoising. It has been proposed that an SWT should be employed for the purpose of noise reduction in order to efficiently identify the defect features present in the vibration signal [23]. The advantages of the SWT over the ordinary WT include the following: SWT provides a more stable frequency domain representation, it facilitates the examination of nonstationary signals, and it is more sensitive to the capture of local details in the signal [24]. Furthermore, the SWT is more appropriate for the study of characteristics such as long-term trends and abrupt changes and, therefore, has a wider application in signal processing and analysis.

The nonlinearity and instability of gearbox vibration signals make the application of linear feature extraction methods impractical. Consequently, entropy-based methodologies grounded in nonlinear dynamics theory have been extensively utilized in electromechanical fault identification. Entropy is an appropriate physical quantity for assessing the regularization procedure and intricacy of data sequences, especially while analyzing nonstationary and nonlinear vibration signals [25]. Reverse dispersion entropy (RDE) [26] and fluctuation dispersion entropy (FDE) [27] represent the improved algorithms of dispersion entropy [28]. RDE redefines the calculation of entropy values with distance information, making it more capable of detecting abrupt signals. Its advantages are more pronounced when dealing with highly impulsive vibration signals compared to traditional entropy algorithms. FDE is designed to accommodate the inherent volatility of the sequence, offering a robust and less susceptible approach to noise interference. Inspired by two improved entropy algorithms, Li et al. [29] proposed fluctuation-based reverse dispersion entropy (FRDE). In contrast to traditional entropy algorithms, FRDE is capable of accurately estimating the complexity of a signal by considering the disparity between neighboring elements within the dataset and by incorporating distance information following the removal of a trend from the data.

However, the aforementioned methods exclusively contemplate signals at a singular scale, which may overlook key time-related data. Costa et al. [30] introduced the concept of multiscale entropy in order to address this constraint. This algorithm effectively addresses the singularity problem of traditional entropy algorithms in expressing timeseries features by applying multiscale coarse-graining processing to the temporal sequence. In recent years, gearbox failure diagnosis technology utilizing multiscale entropy algorithms has seen an increased application. Zheng et al. [31] utilized multiscale fuzzy entropy to extract signal features for fault diagnosis input into support vector machines. Shao et al. [32] improved the entropy bias phenomenon in traditional multiscale entropy algorithms using reverse dispersion entropy, addressing the shortcomings of coarse-graining processing through time-shifted sequences. However, the length of the temporal sequence after multiscale processing is shortened as the scale factor grows, leading to reduced stability of the entropy values. The majority of extant methodologies address this issue through the exclusive utilization of first-order moment processing, thereby failing to consider the potential value of other information present within the timeseries. Therefore, this paper proposes an improved feature extraction method based on ERCMFRDE, extending the ensemble coarse-graining processing method from multiorder moment coarse-grained processing. The attributes of the temporal data can be more comprehensively articulated through the parallel processing of features extracted from multiple perspectives.

It is necessary to classify and identify fault characteristics after extracting them from the vibration signals of the gearbox. The application of neural networks [33] and support vector machines (SVM) [34] to small sample datasets is susceptible to overlearning and to a lack of robust generalization capabilities. The LSSVM [35] is a specific form of SVM that operates under a quadratic loss function. This approach not only simplifies the computational process, but also effectively avoids the characteristics of local minima [36]. Li et al. [37] presented a whole life cycle failure diagnostic technique for high-speed rolling bearings based on enhanced grey wolf optimization for LSSVM. Lu et al. [38] employed a sparse empirical wavelet transform in conjunction with an adaptive dynamic LSSVM for the purpose of failure diagnosis of gear pumps. The particle swarm optimization (PSO) algorithm has the potential to enhance the speed of optimization of LSSVM parameters and augment the overall computational capacity of the model [39]. This paper presents the construction of the PSO–LSSVM model for the purpose of gearbox fault diagnosis.

This paper presents an intelligent diagnostic method for the identification of defects in wind turbine gearboxes based on ICEEMDAN–SWT, ERCMFRDE, and LSSVM. Firstly, the wind turbine gearbox vibration signal is decomposed into noise-containing and noise-free signals by the ICEEMDAN method. The noise-containing components screened with the arrangement entropy as the threshold value are subjected to the SWT noise reduction, and the noise-containing components after noise reduction and the unprocessed noise-free components are reorganized. Secondly, the feature set of the denoised signals is extracted using the ERCMFRDE method, which is based on multiorder central moments. Then, the RFE feature selection is employed to retain the most useful features and the feature set is partitioned into training and testing sets, randomly. Finally, the LSSVM model is then utilized to train the model using the training set, and fault identification is performed on the testing set. The experimental results substantiate the efficacy, superiority, generalizability, and noise resistance of the proposed intelligent diagnosis method.

## 2. Basic Principles

### 2.1. ICEEMDAN–SWT

#### 2.1.1. Rime Optimization Algorithm

RIME simulates the movement of delicate ice crystals for algorithmic search and develops algorithms by replicating the interaction patterns among agents experiencing severe frost conditions.

Phase 1. Soft frost search mechanism:

In breezy conditions, soft frost grows with strong randomness. This enables frost particles to freely cover the surface of the object, but with a gradual growth in a consistent direction. This study presents a novel approach called the soft frost search mechanism, which takes advantage of the high level of randomness and wide coverage provided by frost particles to rapidly cover the entire search space and avoid falling into a local optimal solution.

(1) $$R_{ij}^{new}=R_{best,j}+r_{1}\cdot \cos\theta \cdot \beta \cdot (h\cdot (Ub_{ij}-Lb_{ij})+Lb_{ij}),r_{2}<E\frac{n!}{r!\left(n-r\right)!}$$

The parameters are expressed as:

(2) $$\theta =\pi \cdot \frac{t}{10\cdot T}$$

(3) $$\beta =1-[\frac{w\cdot t}{T}]/w$$

where β is mathematically modeled as a step function, the symbol “[ ]” indicates that rounding is being used, and *w* has a default value of 5 and is utilized to regulate the quantity of segments in the step function. The attachment coefficient, denoted as *E*, directly influences the likelihood of coagulation of the agent and rises as the number of repetitions increases.

(4) $$E=\sqrt{\left(t/T\right)}$$

Phase 2. Hard Frost Perforation mechanism:

In the presence of strong gusty winds, hard frost tends to develop in a more predictable and consistent manner, whereas soft frost is more prone to developing in a more erratic and random manner. Hard frost agents snowball in a single direction, exhibiting a proclivity for crossing phenomena. Consequently, this study presents a hard frost piercing mechanism with the objective of enhancing the convergence of the algorithm and the capacity to escape from local optima.

(5) $$R_{ij}^{new}=R_{best,j},r_{3}<F^{normr}(S_{i})$$

Phase 3. Aggressive greedy selection mechanism:

A positive greedy selection mechanism for population updating in meta-heuristic optimization algorithms. The mechanism decides whether or not to replace an individual by comparing its updated fitness value with its preupdated value and replaces the solutions of both individuals simultaneously.

#### 2.1.2. Improved Complete Ensemble EMD

ICEEMDAN is an enhanced algorithm derived from EMD. The specific steps for its decomposition are as follows:

Step 1. Apply white noise to the original signal *x*:

(6) $$X_{1}^{\left(i\right)}=x+e_{1}E_{1}(w^{\left(i\right)}),i=1,2\cdots n$$

where $e_{1}$ is the noise standard deviation of the first decomposed signal and $w^{\left(i\right)}$ is a series of Gaussian white noises.

Step 2. The first residual:

(7) $$r_{1}=\langle X_{1}^{\left(i\right)}-E_{1}(X_{1}^{\left(i\right)})\rangle$$

where < > indicates averaging.

Step 3. The original signal *x* is subtracted from the first calculation to obtain the residual $r_{1}$ and obtain the first component:

(8) $$IMF\;1=x-r_{1}$$

Step 4. The second residual is estimated as the mean of a series of $r_{1}+e_{2}E_{2}(\omega^{i})$ and the second component is obtained:

(9) $$IMF\;2=r_{1}-r_{2}=r_{1}-\langle (r_{1}+e_{2}E_{2}(w^{i}))\rangle$$

Step 5. The residuals of the $k^{th}$ order modes $r_{k}$ are calculated:

(10) $$r_{k}=\langle X_{k}^{\left(i\right)}-E_{k}(X_{k}^{\left(i\right)})\rangle$$

Step 6. Calculate IMF k.

(11) $$IMF\;k=r_{k-1}-r_{k}$$

where *k* is the total number of IMF.

Step 7. Return to step 5 to calculate $r_{k}$.

#### 2.1.3. Permutation Entropy

**Figure 1:**
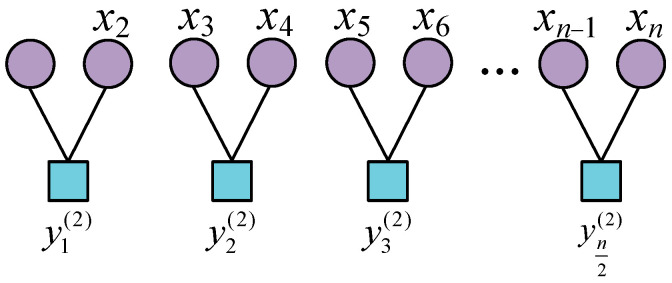
Schematic diagram of the initial signal coarse-graining process.

Assuming that the initial signal sequence is $X=\left\{x_{i}|i=1,2,\ldots ,n\right\}$, the time delay is τ and the embedding parameter is *m*, the steps for calculating the alignment entropy of *X* are as follows:

Step 1. A coarse-graining calculation is performed on the initial signal, and the coarse-graining process is illustrated in Figure 1 when τ = 2.

The sequence of signal reconstruction after coarse graining is represented as Y={y(j)|j=1,2,…,[N/τ]}, where [N/τ] is the rounding parameter. The calculations are as follows:

(12) $$y_{j}^{\tau }=\frac{1}{\tau }\sum_{i=(j-1)\tau +1}^{j\tau }x_{i},1\leq j\leq [N/\tau ]$$

Step 2. The reconstructed sequence is mapped in the phase space under an embedding parameter of *m* on the basis of the signal reconstruction sequence. The equation is as follows:

(13) $$\left[\begin{array}{cccc} y(1) & y(2) & \cdots & y(1+m)\\ y(2) & y(3) & \cdots & y(2+m)\\ \vdots & \vdots & & \vdots \\ y(j) & y(j+1) & \cdots & y(j+m)\\ \vdots & \vdots & & \vdots \\ y(\nu ) & y(\nu +\tau ) & \cdots & y(\nu +m) \end{array}\right]$$

where $\nu =\left[\begin{array}{c} N/\tau \end{array}\right]-m$.

Step 3. The elements in each row vector in the phase space are organized in a decreasing order. The vector of indexed columns is defined as $s(l)=(z_{1},z_{2},\cdots ,z_{m})$ and satisfies l=1,2;⋯ν in order to represent the number of columns in which the elements of each row are organized in a decreasing order. The corresponding descending row vector is denoted as:

(14) $$y(j+z_{1})\leq y(j+z_{2})\leq \cdots \leq y(j+z_{m})$$

There is a total of m! ways of arranging the elements of *s(l)*, and the values of the probability of arranging each row of the phase space are $P_{1},P_{2},\cdots \;,P_{\nu }$, respectively. The value of the entropy of arranging PE is:

(15) $$H_{PE}=-\sum_{j=1}^{m!}P_{j}\ln P_{j}$$

when $P_{j}=1/m!$, the HPE takes the maximum value ln(m!). The HPE is generally normalized:

(16) $$H_{PE}=H_{PE}/\ln(m!)$$

From Equation (16), the HPE belongs to 0~1. The HPE value of regular signals is very small, close to 0, while the entropy of the complex disordered arrangement has a large value, close to 1. Timeseries with PE values greater than 0.6 are typically regarded as noise, whereas timeseries with PE values less than or equal to 0.6 are deemed to be valid components that are strongly correlated with the signal [19].

#### 2.1.4. Stationary Wavelet Transform

SWT exhibits a shift-invariant property in contrast to the WT. Upsampling is utilized in the transformation process rather than employing downsampling. This ensures that the transformed signal retains the same length as the original signal and that the transformed coefficients remain unaltered.

The original signal, designated as f(t), can be expressed in the form of a continuous wavelet transform equation:

(17) $$W_{f}(2^{j},b)=2^{\frac{j}{2}}\int_{-\infty }^{+\infty }f(t)\Psi (\frac{t-b}{2^{j}})dt$$

When b=k, the above equation represents the stationary wavelet transform.

The Mallat decomposition algorithm is:

(18) $$\{\begin{array}{c} a_{j-1,k}=\sum_{n}h_{n}a_{j,2k+n}\\ d_{j-1,k}=\sum_{n}g_{n}a_{j,2k+n} \end{array}$$

The Atrous decomposition algorithm is:

(19) $$\{\begin{array}{c} a_{j-1,k}=\sum_{n}h_{n}a_{j,k+2^{-j}n}\\ d_{j-1,k}=\sum_{n}g_{n}a_{j,k+2^{-j}n} \end{array}$$

where *h* and *g* are low-pass and high-pass filters, respectively. $a_{j-1,k}$ indicates the scale coefficients and $d_{j-1,k}$ indicates the wavelet coefficients, reflecting the approximation signal and wavelet coefficients, respectively.

**Figure 2:**
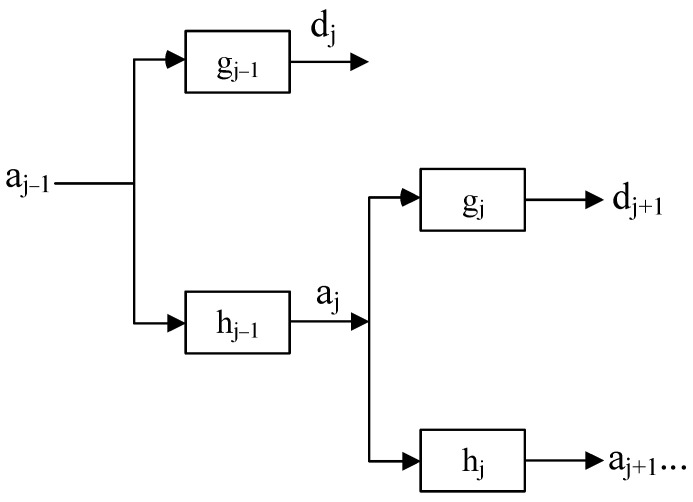
The SWT decomposition process.

The SWT decomposition process is shown in Figure 2.

### 2.2. ERCMFRDE

#### 2.2.1. Fluctuation-Based Reverse Dispersion Entropy

FRDE is a nonlinear dynamic feature extraction method that accurately measures regularities and identifies mutations in vibration signals. For a one-dimensional signal of length *N*: $X=\left\{x_{1},x_{2},\cdots ,x_{N}\right\}$, with the FRDE value of *X* defined as follows [26]:

Step 1. The temporal sequence is normalized and standardized to a range of 0 to 1. We map *X* to $Y=\left\{y_{1},y_{2},\cdots ,y_{N}\right\}$ by normal cumulative distribution function (NCDF).

(20) $$y_{i}=\frac{1}{\sigma \sqrt{2\pi }}\int_{-\infty }^{x_{i}}e^{\frac{-{\left(t-\gamma \right)}^{2}}{2\sigma^{2}}}dt$$

where $y_{i}\in \left(0,1\right)$ and σ and γ represent the standard deviation and average value of the timeseries *X*, respectively.

Step 2. Mapping timeseries *Y* to *c* classes. We map *Y* to $Z^{c}=\left\{z_{1}^{c},z_{2}^{c},\cdots ,z_{N}^{c}\right\}$ by using $round\left(c\cdot y_{i}+0.5\right)$, where *c* represents the number of categories and $z_{i}$ represents a positive integer ranging from 1 to *c*.

Step 3. Reconstruction of the phase space. We perform a reconstruction of *Z* into *T* by using embedding vectors with a time delay of *d* and an embedding dimension of *m*. The matrix comprising all embedding vectors can be written as follows:

(21) $$\left[\begin{array}{l} \left\{z_{1}^{c},z_{1+d}^{c},\cdots ,z_{1+(m-1)d}^{c}\right\}\\ \;\;\;\vdots \;\;\vdots \;\;\;\\ \left\{z_{j}^{c},z_{j+d}^{c},\cdots ,z_{j+(m-1)d}^{c}\right\}\\ \;\;\;\vdots \;\;\vdots \;\;\;\\ \left\{z_{T}^{c},z_{T+d}^{c},\cdots ,z_{T+(m-1)d}^{c}\right\} \end{array}\right]$$

where the number of embedding vectors *T* is equivalent to N−(m−1)d.

Step 4. The mapping of each sequence $z_{j}$ to form a pattern $\pi_{v_{o}v_{1}\cdots v_{m-1}}$ is based on the values inherent to each sequence. The following proposition is, therefore, true:

(22) $$z_{j}^{c}=v_{0},z_{j+d}^{c}=v_{1},z_{j+2d}^{c}=v_{2},\cdots ,z_{j+(m-1)d}^{c}=v_{m-1}$$

The number of potential dispersion patterns that can be associated with each sequence is equal to $(2c-1{)}^{m-1}$.

Step 5. Determining the proportionate occurrence rate of each dispersion pattern. The proportion of occurrences of the $i^{\mathrm{th}}$ dispersion pattern can be mathematically represented as follows:

(23) $$p(\pi_{i})=\frac{Number\left\{\pi_{i}\right\}}{N-(m-1)d}(1\leq i\leq c^{m})$$

where $p(\pi_{i})$ denotes the ratio of the number of $i^{\mathrm{th}}$ dispersion patterns to the number of embedding vectors.

Step 6. Performing FRDE calculations. The expression can be formulated as follows:

(24) $$\mathrm{FRDE}(x,m,c,d)=\sum_{i=1}^{{\left(2c-1\right)}^{m-1}}{\left(p(\pi_{i})-\frac{1}{{\left(2c-1\right)}^{m-1}}\right)}^{2}$$

#### 2.2.2. Ensemble-Refined Composite Multiscale FRDE

In the ERCMFRDE framework, the multiple temporal sequence *x* is generated with distinct starting points for the coarse-graining process, using a scale factor *τ*. The $k^{th}$ coarse-grained timeseries $x_{k}^{\left(\tau \right)}=\left\{x_{k,1}^{\left(\tau \right)},x_{k,2}^{\left(\tau \right)},\cdots ,x_{k,j}^{\left(\tau \right)}\right\}$ of *u* at the scale level *τ* can be calculated by employing a variety of distinct methodologies to generate multiple coarse-grained series. 

The main coarse-grained treatments of the first-order moment method are calculated as follows:

(25) $$x_{k,j}^{\left(\tau \right)}{|}_{mean}=\frac{1}{\tau }\sum_{b=k+\tau (j-1)}^{k+\tau j-1}u_{b},1\leq j\leq N,1\leq k\leq \tau$$

(26) $$x_{k,j}^{\left(\tau \right)}{|}_{\max}=\underset{k+\tau (j-1)\leq b\leq k+\tau j-1}{\max}(u_{b}),1\leq j\leq N,1\leq k\leq \tau$$

(27) $$x_{k,j}^{\left(\tau \right)}{|}_{\min}=\underset{k+\tau (j-1)\leq b\leq k+\tau j-1}{\min}(u_{b}),1\leq j\leq N,1\leq k\leq \tau$$

The main coarse-grained treatments of the second-order moment method are calculated as follows:

(28) $$x_{k,j}^{\left(\tau \right)}{|}_{\mathrm{var}}=\frac{1}{\tau }{\sum_{b=k+\tau (j-1)}^{k+\tau j-1}\left(u_{b}-{\bar{u}}_{b,j}\right)}^{2},1\leq j\leq N,1\leq k\leq \tau$$

(29) $$x_{k,j}^{\left(\tau \right)}{|}_{rms}=\sqrt{\frac{1}{\tau }\sum_{b=k+\tau (j-1)}^{k+\tau j-1}{u_{b}}^{2}},1\leq j\leq N,1\leq k\leq \tau$$

where ${\bar{u}}_{b,j}=\frac{1}{\tau }\sum_{b=k+\tau (j-1)}^{k+\tau j-1}u_{b}$.

The main coarse-grained treatment of the third-order moment method is calculated as follows:

(30) $$x_{k,j}^{\left(\tau \right)}{|}_{skewness}=\frac{1}{\tau }{\sum_{b=k+\tau (j-1)}^{k+\tau j-1}\left(u_{b}-{\bar{u}}_{b,j}\right)}^{3},1\leq j\leq N,1\leq k\leq \tau$$

where ${\bar{u}}_{b,j}=\frac{1}{\tau }\sum_{b=k+\tau (j-1)}^{k+\tau j-1}u_{b}$.

For different coarse-grained series, the following definitions apply to the RCMFRDE values of different coarse-grained processing:

(31) $$\mathrm{RCMFRDE}\_t(x,c,m,d,\tau )=\frac{1}{\tau }\sum_{k=1}^{\tau }\mathrm{FRDE}(x_{k,j}^{\left(\tau \right)}{|}_{t},c,m,d)$$

where *t* refers to the type of coarse graining, including mean, max, min, var, rms, and skewness.

The RCMFRDE of ensemble first-order moment coarsening (ERCMFRDE_1) is constituted of the RCMFRDE of mean processing (RCMFRDE_mean), the RCMFRDE of maximum coarse-grained processing (RCMFRDE_max), and the RCMFRDE of minimum coarse-grained processing (RCMFRDE_min):

(32) ERCMFRDE_1=[RCMFRDE_mean;RCMFRDE_max;RCMFRDE_min]

The RCMFRDE of ensemble second-order moment coarsening (ERCMFRDE_2) is constituted of the RCMFRDE of variance coarse-grained processing (RCMFRDE_var) and the RCMFRDE of root mean square coarse-grained processing (RCMFRDE_rms):

(33) ERCMFRDE_2=[RCMFRDE_var;RCMFRDE_rms]

As the third-order coarse-graining encompasses solely the skewness treatment, the RCMFRDE of the ensemble third-order moment coarsening (ERCMFRDE_3) is identical to the RCMFRDE of the skewness coarse-graining treatment (RCMFRDE_skewness).

(34) ERCMFRDE_3=RCMFRDE_skewness

The ERCMFRDE is constituted of ERCMFRDE_1, ERCMFRDE_2, and ERCMFRDE_3(RCMFRDE ensemble of six coarse-grained treatments):

(35) ERCMFRDE=[ERCMFRDE_1;ERCMFRDE_2;ERCMFRDE_3]

#### 2.2.3. Recursive Feature Elimination

The ERCMFRDET feature set contains a large number of features, many of which may be superfluous. These redundant features can lead to overfitting of the algorithm model, reducing its generalization ability. Feature selection is a common preprocessing step that has the effect of enhancing model performance, reducing computational costs, and accelerating the training process.

**Figure 3:**
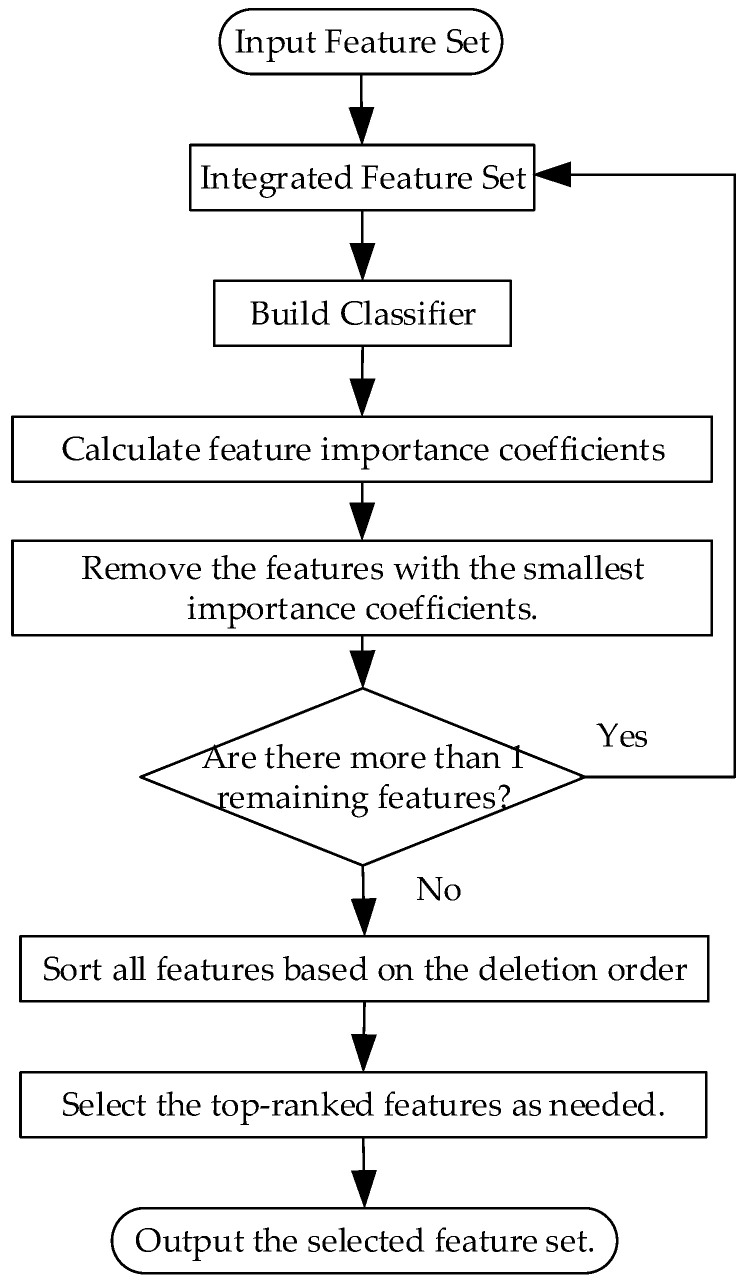
The flowchart of the RFE algorithm.

RFE [40] is a bottom-up method that starts with all features and iteratively construct models while it eliminates the least important features to choose the most advantageous set of features. In each iteration, RFE trains a model and uses feature importance scores (such as coefficient magnitude, information gain, etc.) to determine which features should be removed. This procedure is iterated until the necessary quantity of characteristics is attained or until a predetermined termination condition is satisfied. RFE can be used with different evaluation methods and models, making it highly flexible and applicable. Moreover, RFE demonstrates good stability and reliability in feature selection. Figure 3 shows the flowchart of the recursive feature elimination algorithm.

### 2.3. Least Squares Support Vector Machine

LSSVM represents a significant advancement in SVM theory, offering a sophisticated and comprehensive theoretical framework. It is capable of converting the solution to a quadratic optimization problem into the solution to a system of linear equations, thereby streamlining the problem-solving process. Consequently, it has been effectively implemented in a number of fields.

Assuming a training set $\left(x_{i},y_{i}\right)$, where $x_{i}=(x_{1},x_{2},\ldots ,x_{n})$ is the d-dimensional input vector, $y_{i}$ represents the corresponding output data and *n* represents the total number of training data points. The employed nonlinear function estimation is modeled in the following form:

(36) f(x)=b+〈ϕ(x),w〉

where *w* is the weight vector, *b* is the bias term, and the symbol < > refers to the inner product operation.

The assessment problem is described as an optimization problem based on the structured risk minimization principle:

(37) $$\begin{array}{c} \min J(w,e)=\min(\frac{1}{2}w^{2}+\frac{1}{2}\gamma \sum_{i=1}^{N}e_{i}^{2})\\ s.t.\;y_{i}=w,\phi (x_{i})+b+e_{i}\;i=1,2,\ldots ,N\\ \gamma >0 \end{array}$$

The regularization parameter γ is used to identify the optimal balance between model complexity and accuracy. The regression error $e_{i}$ represents the discrepancy between the observed and predicted values of the output.

The Lagrange function is formulated to represent the solution to resolve the aforementioned optimization issue.

(38) $$L_{\mathrm{LSSVM}}=\frac{1}{2}w^{2}+\frac{1}{2}\gamma \sum_{i=1}^{N}e_{i}^{2}-\sum_{i=1}^{N}\alpha_{i}\left\{w,\phi (x_{i})+b+e_{i}-y_{i}\right\}$$

where $\alpha_{i}$ represents the Lagrange multiplier.

The optimal solution to the problem can be obtained by setting the derivatives of *w*, *b*, $e_{i}$, and $\alpha_{i}$ to zero.

(39) $$\frac{\partial L_{\mathrm{LSSVM}}}{\partial w}=0\to w=\sum_{i=1}^{N}\alpha_{i}\phi (x_{i})$$

(40) $$\frac{\partial L_{1.\mathrm{SSVM}}}{\partial b}=0\to \sum_{i=1}^{N}\alpha_{i}=0$$

(41) $$\frac{\partial L_{L.\mathrm{SSVM}}}{\partial e_{i}}=0\to \alpha =\gamma e_{i}$$

(42) $$\frac{\partial L_{1.\mathrm{SSVM}}}{\partial \alpha_{i}}=0\to <w,\phi (x_{i})>+b+e_{i}-y_{i}=0$$

The four linear problems can be reduced to a single equation by eliminating *w* and $e_{i}$.

(43) $$\left[\begin{array}{cc} 0 & E^{T}\\ E & \Omega +\frac{1}{\gamma }E \end{array}\right]\left[\begin{array}{c} b\\ \alpha \end{array}\right]=\left[\begin{array}{c} 0\\ y \end{array}\right]$$

where $y={\left(y_{1},y_{2},\ldots ,y_{n}\right)}^{T}$, $\alpha ={\left(\alpha_{1},\alpha_{2},\ldots ,\alpha_{n}\right)}^{T}$, *E* is the identity matrix and Ω is a symmetric matrix of N × N kernel functions:

(44) $$\Omega_{ij}=K(x_{i},x_{j})=\phi {\left(x_{i}\right)}^{T}\phi (x_{j})\;i,j=1,2,\cdots ,N$$

where $K(x_{i},x_{j})$ is the kernel function that satisfies Meser’s condition.

Then, the LSSVM model can be expressed as:

(45) $$\begin{array}{c} y(x)=w,\phi (x)+b\\ =\sum_{i=1}^{n}\alpha_{i}\phi (x_{i})\cdot \phi (x)+b\\ =\sum_{i=1}^{n}\alpha_{i}K(x_{i},x)+b \end{array}$$

The radial basis function (RBF) kernel is a commonly employed function in various fields of study. The mathematical expression is as follows:

(46) $$K(x_{i},x_{j})=\exp\left(-\frac{x_{i}-{x_{j}}^{2}}{2\sigma^{2}}\right),\sigma >0$$

where σ is the bandwidth of the kernel function.

The two hyperparameters, γ and σ, are crucial parameters that significantly influence the performance of the LSSVM model and require meticulous determination. As evidenced in the literature [39], the PSO algorithm has the capacity to accelerate the optimization of LSSVM parameters and enhance the overall computational efficacy of the model. Consequently, this study has selected the PSO algorithm to optimize the model parameters.

## 3. The Failure Diagnostic Flowchart

**Figure 4:**
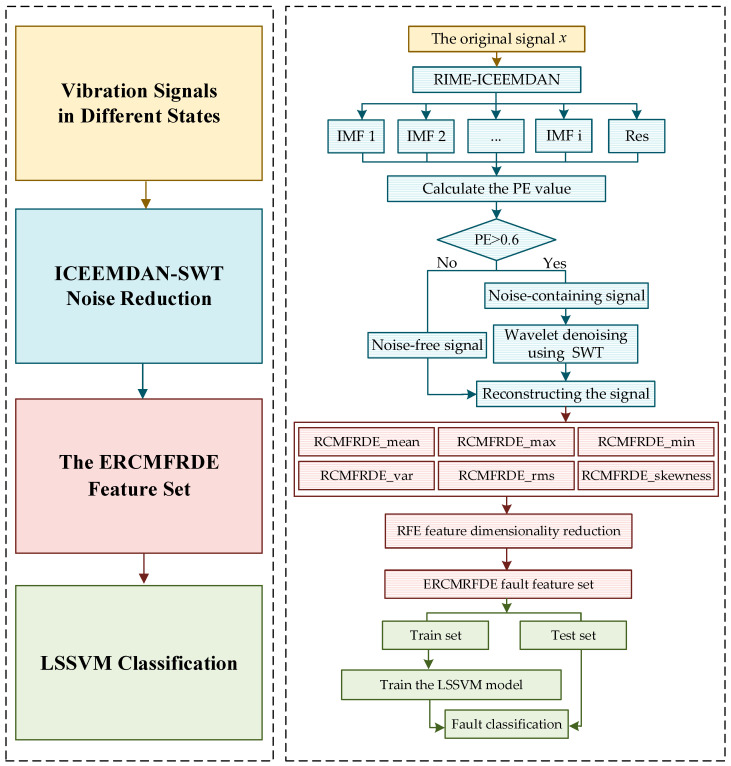
The fault diagnosis flowchart.

To address the issues of nonstationarity, nonlinearity, and noise in wind turbine gearbox vibration signals, this paper proposes a gear failure diagnostic method based on ERCMFRDE, combined with ICEEMDAN–SWT, RFE, and LSSVM. Figure 4 displays the failure diagnostic flowchart. The precise sequence of steps is as follows:

Step 1. The utilization of acceleration sensors enables the acquisition of vibration signals during the operational process of the gearbox.

Step 2. The RIME algorithm is employed for the purpose of optimizing two critical parameters of ICEEMDAN, namely Nsd and NR. The noise signal is decomposed using the ICEEMDAN model with optimized parameters to obtain a series of IMF components and a Res component.

Step 3. The PE value for each IMF component is calculated. SWT is employed to reduce noise in the case of the IMF components with a PE value exceeding 0.6 [19]. The remaining IMF components are retained in their original form. All the IMF components after noise reduction and those not involved in noise reduction are recombined to obtain the post-noise reduction signal.

Step 4. The RCMFRDE values of the signals are calculated as the feature set of the signals, resulting in a subset of fault features based on six different coarse-grained processes.

Step 5. The feature set integrated with six subsets of fault features is subjected to feature dimensionality reduction using the recursive feature elimination algorithm, selecting the top 20 feature values. These selected features form a new fault feature set.

Step 6. The fault feature set is partitioned into training and testing sets, randomly, based on a predefined ratio. The LSSVM model is then utilized to train the model using the training set. Subsequently, fault identification is performed on the testing set.

## 4. Experimental Verification

In this paper, the fault signals are extracted by simulating the gearbox operation state on the mechanical failure simulation (MFS) experimental platform, and the intelligent diagnosis process is carried out on the software MATLAB R2022b.

### 4.1. Signal Acquisition and Noise Reduction

**Figure 5:**
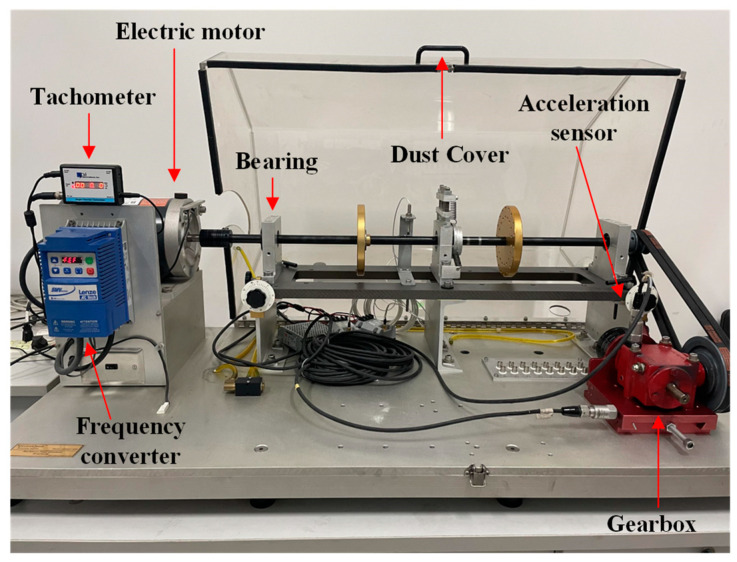
MFS experimental platform.

**Figure 6:**
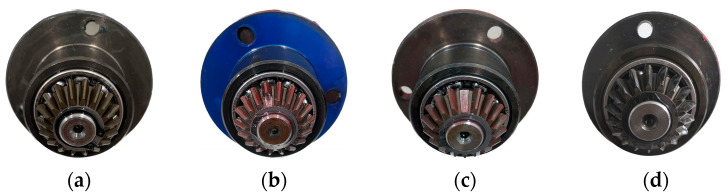
Four states of the gear: (**a**) normal state; (**b**) broken tooth fault; (**c**) missing tooth fault; (**d**) surface wear fault.

The dataset pertaining to the gearbox was obtained through the utilization of an MFS experimental platform. The central part of the experimental system consisted of a driving motor, bearings, gearbox, and the data acquisition equipment, as shown in Figure 5. The motor speed could reach up to 3600 rpm. Additionally, a magnetic powder brake attached to the output terminal of the gearbox allowed for the manual adjustment of the corresponding load state by rotating it. The scale displayed values from 0 to 5, corresponding to torque values ranging from 0.056 Nm to 1.129 Nm. During the operation of the experimental setup, the accelerometers captured vibration data from both the gearbox and its base, with signals sampled at a frequency of 15 kHz. This experimental setup enabled the analysis of the vibrational properties of individual equipment faults and the investigation of the coupling effects between multiple faults by combining different fault modules during mechanical operation simulation. As illustrated in Figure 6, the gearbox fault diagnostic research toolkit employed in the experiments encompassed normal state (NOR) gears, broken tooth fault (BTF) gears, missing tooth fault (MTF) gears, and surface wear fault (SWF) gears.

The studies were carried out at a motor speed of 1750 rpm to evaluate the motor’s normal condition as well as three different fault situations. The initial sample for each state was divided into 100 subsamples, each containing 2048 sampling points. Each set of subsamples was further split into training and testing sets following a 3:2 ratio (See Table 1).

**Figure 7:**
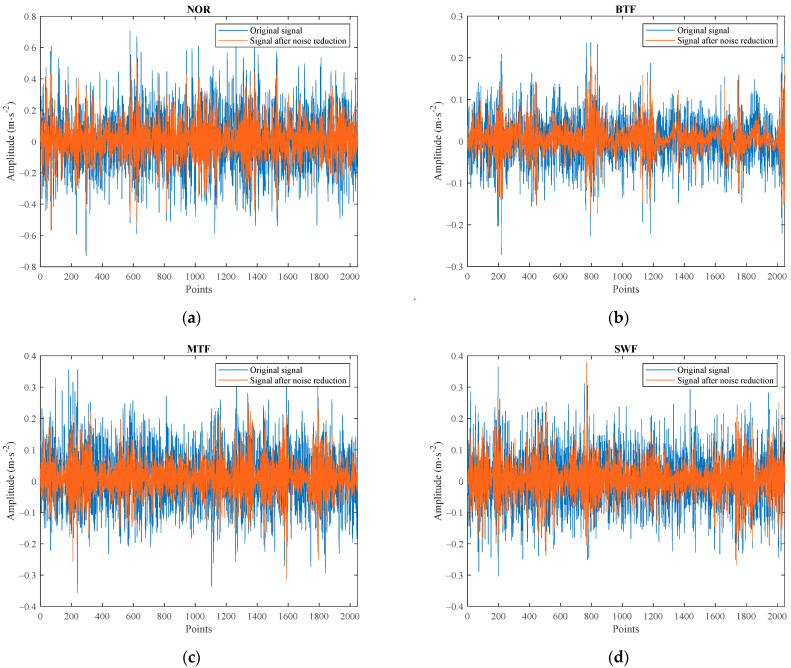
Original signal and denoised signal. (**a**) Normal state, (**b**) broken tooth fault, (**c**) missing tooth fault, (**d**) surface wear fault.

The acquired vibration signals of the four faulty gears were subjected to the ICEEMDAN–SWT denoising process, and the obtained vibration waveform are shown in Figure 7. It can be observed that the noise-canceled signal exhibited more pronounced vibration shocks compared to the original signal, indicating that the ICEEMDAN–SWT noise reduction method can improve the signal quality and retain useful fault characteristic information.

### 4.2. Feature Extraction

**Figure 8:**
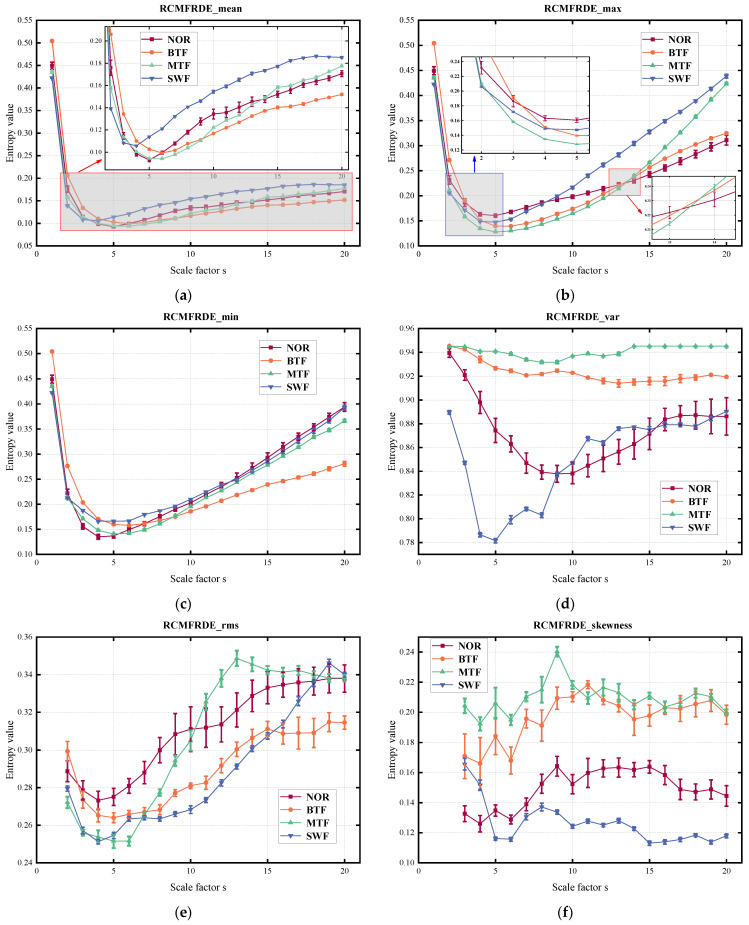
The RCMFRDE value. (**a**) RCMRDE_mean, (**b**) RCMFRDE_max, (**c**) RCMRDE_min, (**d**) RCMFRDE_var, (**e**) RCMRDE_rms, and (**f**) RCMFRDE_skewness.

The RCMFRDE values with different coarse-grain processing of each signal in the sample set were computed under the following parameters: embedding dimension *m* = 2, scale factor *s* = 20, classification category *c* = 6, and time delay *d* = 1 [25]. This resulted in six fault feature matrices, shown in Table 2. Figure 8 shows the error bar plots of RCM–FRDE computed for the four gear states.

It can be observed that the RCMFRDE values yielded distinct fault features based on different coarse-graining theories from the error bar plots. By comparing the first three graphs in Figure 8 with the last three graphs, the error bars of (a), (b), and (c) appear relatively small, indicating a higher stability of the entropy values when the coarse-graining mode was a first-order moment. The comparison of (a), (b), and (c) in Figure 8 illustrates that the RCMFRDE_mean and RCMFRDE_max showed good discrimination on the vast majority of scales, but RCMFRDE_min was not practical for discriminating between NOR and SWF. As shown in Figure 8d,e, the trends of RCMFRDE after two different second-order moment coarsening treatments were also different. RCMFRDE_var exhibited higher discrimination at most scales, but distinguishing between NOR and SWF became challenging at more prominent scale factors. However, the characteristic curves of RCMFRDE_rms for different faults overlapped at more scales. In Figure 8f, clear distinctions in entropy curves for each fault state were observed when the scale factor was between 5 and 15.

In general, RCMFRDE from different coarse-grained processing was not consistently discriminated at different scales. However, ensemble processing enabled features to complement each other, thereby enhancing feature differentiation. ERCMFRDE-based feature extraction leveraged different characteristics to extract comprehensive feature information from timeseries data, providing a more comprehensive approach to fault feature extraction compared to single-fine-scale composite multiscale entropy extraction methods.

### 4.3. Feature Selection

**Figure 9:**
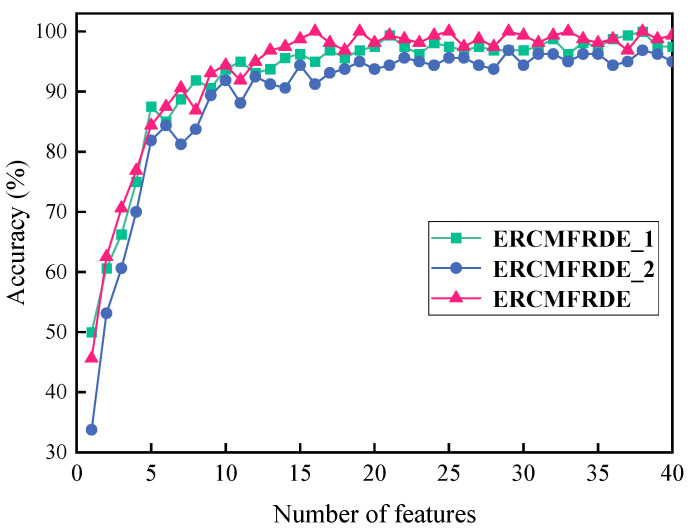
Relationship among the number of different features and accuracy.

As illustrated in Table 2, six distinct feature sets were obtained following the extraction of fault features for all types of faults using RCMFRDE. The implementation of four distinct ensemble processes yielded four ERCMFRDE feature sets, as illustrated in Table 3. Unsupervised feature selection using RFE was implemented for feature importance in ERCMFRDE_1, ERCMFRDE_2, and ERCMFRDE, respectively (given that ERCMFRDE_3 comprised solely RCMFRDE_skewness and possessed a limited number of features, it was unnecessary to apply feature selection.) Subsequently, the significance of RFE sorting of the features was investigated, with the different number of features after RFE sorting input into PSO–LSSVM for fault classification. Due to the large number of features, the author calculated the accuracy rate for the number of features within 40, with the results presented in Figure 9.

Figure 9 indicates that, when the number of features exceeded 20, the accuracy of ERCMFRDE_1, ERCMFRDE_2, and ERCMFRDE became saturated. Consequently, the top 20 most crucial features were selected as the set of features to be employed for fault classification. For the three ensemble feature sets after feature selection, the size of each feature set was 400 × 20. Furthermore, the ERCMFRDE method exhibited the lowest number of requisite features to achieve optimal accuracy and the highest classification accuracy at saturation, thereby demonstrating its superiority over other ensemble processing.

### 4.4. Intelligent Diagnosis

**Figure 10:**
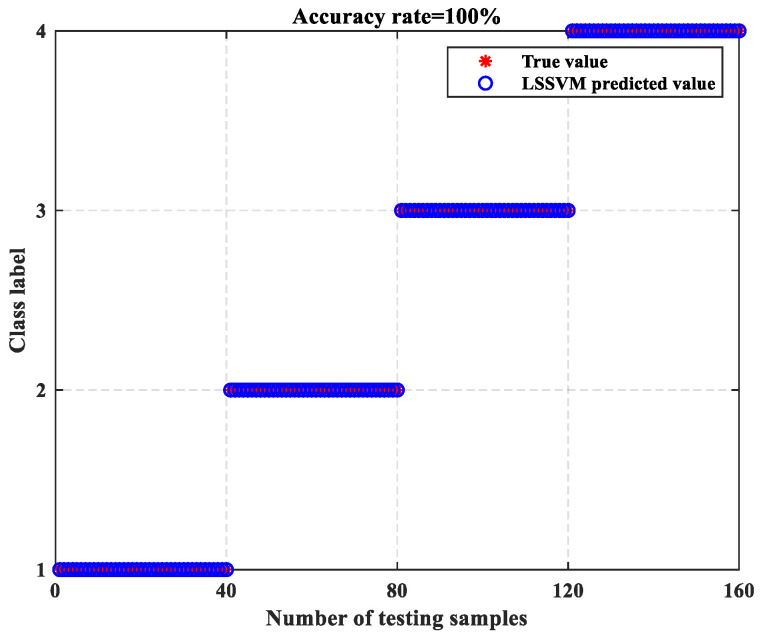
The LSSVM classification results.

The ERCMFRDE feature set was then partitioned into training and testing samples, with the training samples used to train the parameters of PSO–LSSVM and the testing samples fed into the trained LSSVM for fault classification. The findings of the fault diagnosis are displayed in Figure 10, with a fault diagnosis accuracy of 100% and no misclassification samples.

We applied feature extraction algorithms using RCMFRDE with different coarse-graining treatments as well as RCMFRDE with different ensemble processing. Twenty repeated experiments were conducted using the same fault sample data and the aforementioned feature extraction methods to compare different feature extraction algorithms and verify the advantage of ERCMFRDE in comprehensively extracting signal features.

**Figure 11:**
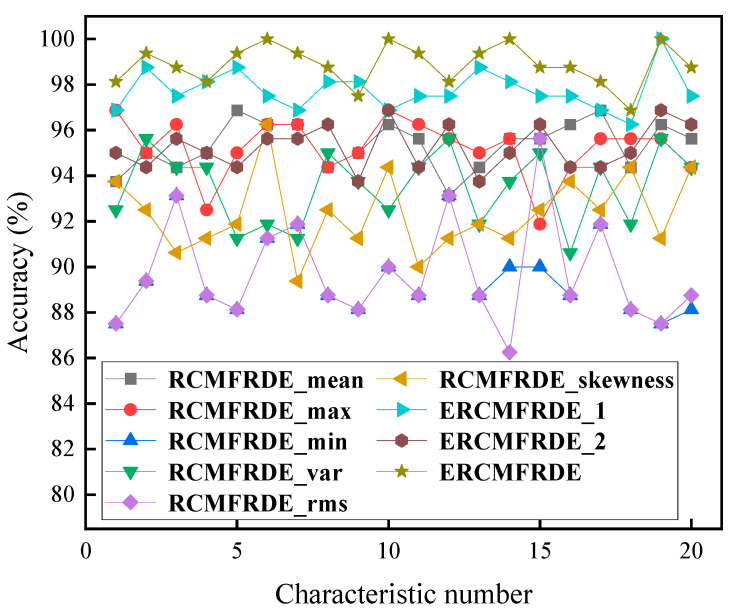
Classification results of RCMFRDE with different coarse-grained processing.

The fault diagnosis accuracy rates of nine feature extraction algorithms for 20 repeated experiments are shown in Figure 11, and the maximum, minimum, and average fault diagnosis accuracy rates are shown in Table 4. It can be observed that the ERCMFRDE algorithm, which employs a comprehensive signal feature extraction process, achieved the highest accuracy in fault diagnosis, with an average diagnosis accuracy that was superior to that of the other eight algorithms. The experiment demonstrated that the multiscale fluctuation-based reverse dispersion entropy algorithm, integrated with multiple coarse-graining processes, provide more practical information in feature extraction.

### 4.5. Noise Resistance Test

In practical engineering applications, the data collected by vibration acceleration sensors installed in wind turbine gearboxes often contain large amounts of noise. We conducted experiments on the fault diagnosis of gearboxes in a noisy background in order to explore the robustness of the model and verify the practicality and anti-interference ability of the model in a real environment.

**Figure 12:**
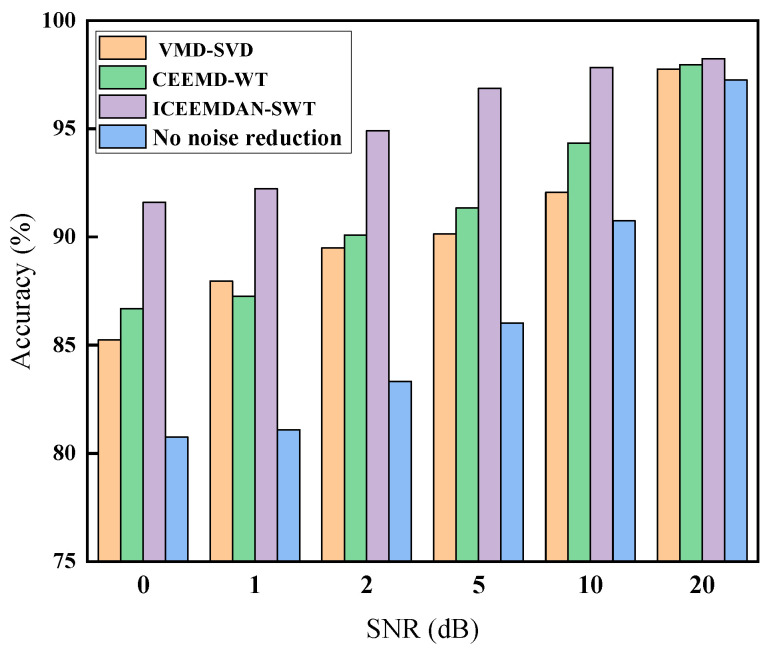
Classification results of different models with noise content.

Gaussian white noise with different levels of interference was added to the dataset to achieve different signal-to-noise ratios (SNR) of 0 dB, 1 dB, 2 dB, 5 dB, 10 dB, and 20 dB. We used five sets of noisy signals to validate the performance of the ICEEMDAN–SWT model proposed in this study regarding noise immunity, and selected VMD–SVD, CEEMD–WT, and no noise reduction as the control benchmark models for the comparison experiments. Each group of experiments was performed 20 times and the average value was calculated, and the results are presented in Figure 12.

In a noise environment with an SNR of 10 dB or 20 dB, the model was largely unaffected by noise in its judgement of faults. In a strong noise environment with an SNR of 0 dB, it still maintained high diagnostic classification accuracy. The diagnostic classification accuracy of the other network models decreased dramatically in the case of increasing noise. It can be concluded that the ICEEMDAN–SWT model proposed in this study exhibits superior anti-noise performance in comparison to other models.

### 4.6. Comparison Experiment

#### 4.6.1. Comparison of Different Entropy Algorithms

We compared ERCMFRDE with widely used entropy-based feature extraction methods to demonstrate the superiority of the FRDE method. We compared it with the ensemble-refined composite multiscale DE (ERCMDE), ensemble-refined composite multiscale RDE (ERCMRDE), and ensemble-refined composite multiscale FDE (ERCMFDE). The parameters of the various entropy algorithms were chosen consistently and as shown below: *l* = 2048, *s* = 20, *d* = 1, *m* = 2, *c* = 6.

**Figure 13:**
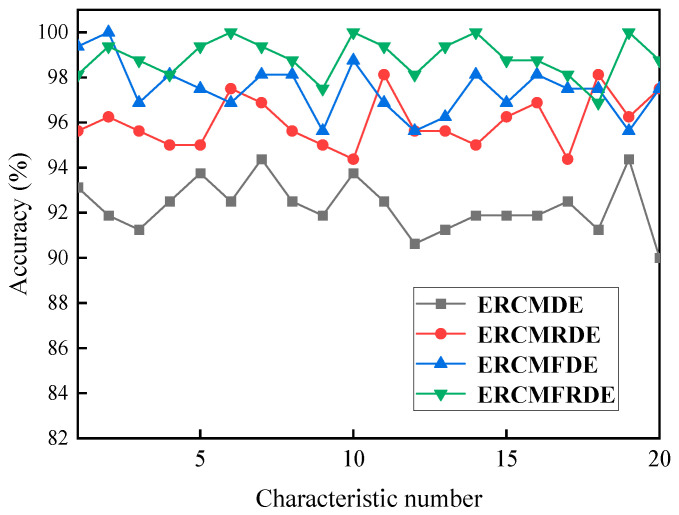
Classification results of different entropy algorithms.

The above entropy methods were used to extract the feature set of the fault signal after noise reduction, and the feature matrix was fed into the LSSVM classification model to train and predict the classification results. The intelligent classification was repeated 20 times, and the average accuracy of the fault diagnosis, as well as the maximum and minimum accuracy values, are presented in Table 5. Figure 13 demonstrates the accuracy of the different entropy algorithms for each classification result.

As shown in Figure 13, the proposed ERCMFRDE for gearbox fault diagnosis yielded an average improvement of 1.41%, 2.85%, and 6.6% compared to the other three feature entropy algorithms. Furthermore, the maximum and minimum values were larger than those of the other feature entropy algorithms. The results indicate that the FRDE features were more effective at addressing timeseries problems and were more sensitive to gearbox state information than FDE, RDE, and DE.

#### 4.6.2. Comparison of Different Diagnostic Methods

In the comparative experiments presented in this section, gearbox vibration datasets from two different sources were selected as raw data: Data1 and Data2. Data 1 came from the gearbox vibration fault data extracted from the MFS fault platform in Section 4.1.

Data 2 was derived from the gearbox failure dataset published by Prof. Jiong Tang’s team at the University of Connecticut, USA. The experimental platform is described in the literature [41]. The dataset tested gears in nine states, including healthy condition, missing tooth, root crack, spalling, and chipping tip with five different levels of severity. The dataset comprised 100 samples for each of the nine types of vibration signals, resulting in a total of 900 samples, each of which was 3600 samples in length.

**Figure 14:**
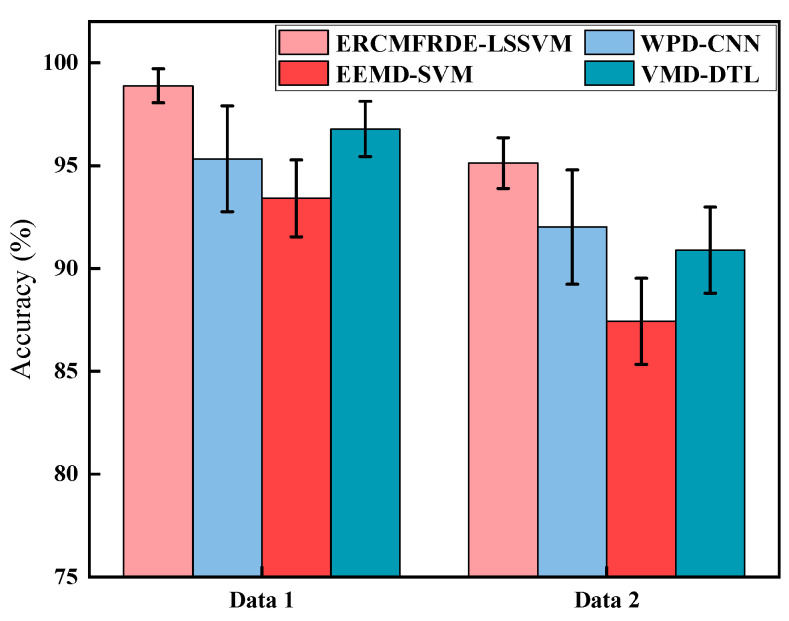
Classification results of different diagnosis methods.

In order to demonstrate the superiority of the proposed ERCMFRDE–LSSVM method, in this paper, we compared it with three classical gearbox fault diagnosis methods: WPD–CNN [42], EEMD–SVM [43], and VMD-DTL [44], based on two data sources. The models trained under different methods were subjected to 20 iterations of random testing of the prediction set. The mean and standard deviation of the 20 recognition accuracies were then calculated and used as the final fault diagnosis results, as illustrated in Figure 14.

As illustrated in Figure 14, the ERCMFRDE–LSSVM method demonstrated a markedly superior success rate in diagnosing gearbox faults across two distinct datasets, outperforming the other three classic methods. This highlights its notable efficiency and superiority. Furthermore, the proposed method demonstrated a low error rate and minimal fluctuations in 20 diagnostic results, indicating its capacity to adapt to diverse vibration signal characteristics and failure modes with relative stability. This observation reflects the method’s stability and reliability.

## 5. Conclusions

This paper proposes a novel intelligent failure diagnostic method for gearboxes based on ICEEMDAN–SWT, ERCMFRDE, and LSSVM, targeting the characteristics of wind turbine gearbox vibration signals and working environment. Initially, the vibration signal from the wind turbine gearbox was denoised by the ICEEMDAN–SWT noise reduction model. Subsequently, the ERCMFRDE values of the denoised signal were extracted and fed into the LSSVM model for training and identification. The conclusions obtained are as follows:

1. A comparison of the ERCMFRDE-based feature extraction method with other feature extraction methods revealed that the ERCMFRDE method had the highest accuracy rate, proving to be significantly better than the entropy algorithm with a single coarse-grained processing and the traditional kinds of entropy algorithms. This evidence serves to illustrate the superiority of the proposed ensemble entropy method for feature extraction and fault identification.
2. In a noisy environment, the fault diagnosis method based on ICEEMDAN–SWT and ERCMFRDE continued to perform well, indicating that the method is effective in eliminating interference components unrelated to fault characteristics. This indicates that the method displays both excellent generalization capabilities and robust performance.
3. The efficacy and reliability of the fault diagnosis methodology based on ERCMFRDE were verified using two gearbox datasets from different sources. The method could accurately identify the type of faults in gearboxes and has more significant advantages over the other three classic fault diagnosis methods.
4. The fault diagnosis method based on ERCMFRDE proposed in this paper is a practical approach for extracting the fault features of gearboxes and accurately identifying different types, with an accuracy of 98.88%. This demonstrates that the proposed intelligent diagnostic method offers a significant advantage in gearbox fault diagnosis.
5. The methods and models investigated in this paper are mainly data-driven and lack consideration of fault mechanisms and knowledge of the unit operating mechanisms. In future research, the complex operating mechanisms of wind turbine gearboxes will be considered and embedded in the data-driven model to guide feature and model learning.

## Figures and Tables

**Table 1:** Description of the MFS gearbox dataset.

Fault Types	Motor Speed (r/min)	Number of Training Samples	Number of Testing Samples	Class Label
Normal state	1750	60	40	1
Broken tooth	1750	60	40	2
Missing tooth	1750	60	40	3
Surface wear	1750	60	40	4

**Table 2:** The results in six fault feature matrices.

Entropy Value	Moment Processing	Coarse Graining	Size
RCMFRDE_mean	First-order	Mean	400 × 20
RCMFRDE_max	First-order	Maximum	400 × 20
RCMFRDE_min	First-order	Minimum	400 × 20
RCMFRDE_var	Second-order	Variance	400 × 19
RCMFRDE_rms	Second-order	Root mean square	400 × 19
RCMFRDE_skewness	Third-order	Skewness	400 × 18

**Table 3:** The results in four fault feature matrices.

Ensemble Entropy Set	Entropy Value	Moment Processing	Coarse Graining	Size
ERCMFRDE_1	RCMFRDE_mean	First-order	Mean	400 × 60
	RCMFRDE_max		Maximum	
	RCMFRDE_min		Maximum	
ERCMFRDE_2	RCMFRDE_var	Second-order	Variance	400 × 38
	RCMFRDE_rms		Root mean square	
ERCMFRDE_3	RCMFRDE_skewness	Third-order	Skewness	400 × 18
ERCMFRDE	RCMFRDE_mean	First-order	Mean	400 × 116
	RCMFRDE_max		Maximum	
	RCMFRDE_min		Maximum	
	RCMFRDE_var	Second-order	Variance	
	RCMFRDE_rms		Root mean square	
	RCMFRDE_skewness	Third-order	Skewness	

**Table 4:** Diagnostic accuracy of nine kinds of algorithm.

Entropy Value	Accuracy (%)		
	Maximum	Minimum	Average
RCMFRDE_mean	96.88	93.13	95.34
RCMFRDE_max	96.88	91.88	95.22
RCMFRDE_min	93.13	87.50	89.59
RCMFRDE_var	95.63	90.63	93.50
RCMFRDE_rms	95.63	86.25	89.72
RCMFRDE_skewness	96.25	89.38	92.34
ERCMFRDE_1	100.00	96.25	97.75
ERCMFRDE_2	96.88	93.75	95.25
ERCMFRDE	100.00	96.88	98.88

**Table 5:** Diagnostic accuracy of four kinds of entropy algorithm.

Entropy Value	Accuracy (%)		
	Maximum	Minimum	Average
ERCMDE	94.38	90.00	92.28
ERCMRDE	98.13	94.38	96.03
ERCMFDE	100.00	95.63	97.47
ERCMFRDE	100.00	96.88	98.88

## Data Availability

The data presented in this study are available upon request from the corresponding author.

## Abbreviations

The following abbreviations are used in this manuscript:

DE	Dispersion Entropy
RDE	Reverse Dispersion Entropy
FDE	Fluctuation Dispersion Entropy
FRDE	Fluctuation-based Reverse Dispersion Entropy
ERCMDE	Ensemble Refined Composite Multiscale DE
ERCMRDE	Ensemble Refined Composite Multiscale RDE
ERCMFDE	Ensemble Refined Composite Multiscale FDE
RCMFRDE	Refined Composite Multiscale Reverse Dispersion Entropy
ERCMFRDE	Ensemble RCMFRDE
EMD	Empirical Mode Decomposition
EEMD	Ensemble EMD
CEEMD	Complementary EEMD
ICEEMDAN	Improved CEEMD with Adaptive Noise
WT	Wavelet Transform
SWT	Stationary Wavelet Transform
PE	Permutation Entropy
IMF	Intrinsic Modal Function
SVM	Support Vector Machine
LSSVM	Least Squares Support Vector Machine
RIME	Rime Optimization Algorithm
Nsd	Noise standard deviation
NR	Number of Realizations
NOR	Normal State
MTF	Missing Tooth Fault
BTF	Broken Tooth Fault
SWF	Surface Wear Fault
PSO	Particle Swarm Optimization
rms	Root Mean Square
MFS	Mechanical Failure Simulation
SNR	Signal-to-Noise Ratio
DTL	Deep Transfer Learning
VMD	Variational Mode Decomposition
WPD	Wavelet Packet Decomposition
CNN	Convolutional Neural Network
